# The impact of hemodialysis schedules on the day of the week of hospitalization for cardiovascular and infectious diseases, over a period of 20 years

**DOI:** 10.1371/journal.pone.0180577

**Published:** 2017-07-10

**Authors:** Masataka Banshodani, Hideki Kawanishi, Shingo Fukuma, Misaki Moriishi, Sadanori Shintaku, Shinichiro Tsuchiya

**Affiliations:** 1 Department of Artificial Organs, Akane-Foundation, Tsuchiya General Hospital, Hiroshima, Japan; 2 Faculty of Medicine, Hiroshima University, Hiroshima, Japan; 3 Human Health Sciences, Graduate School of Medicine, Kyoto University, Kyoto, Japan; The University of Tokyo, JAPAN

## Abstract

There have been no studies as yet that have evaluated how hemodialysis (HD) schedules affect the day of the week of hospitalization for cardiovascular diseases (CVDs) and infectious diseases (IDs), over a period of time. Herein, we performed a retrospective observational cohort study (1995–2014) evaluating 11,111 hospitalizations in 1,953 patients with end-stage renal disease, receiving HD 3 times a week (following either a Monday-Wednesday-Friday [MWF] schedule or a Tuesday-Thursday-Saturday [TTS] schedule) or receiving frequent HD (FHD) at least 4 times a week. Overall, hospitalization rates for CVDs and IDs were the highest on Monday in the MWF schedule and Tuesday in the TTS schedule compared to the average rates for all the days of the week. When generalized estimating equations (GEEs) were used in conjunction with robust variance estimators of each type of CVD, the risk for pulmonary edema was found to be significantly higher on Sunday and Monday in the MWF schedule and Monday and Tuesday in the TTS schedule. For both cerebrovascular and ischemic heart disease, the risks were significantly higher on Tuesday in the MWF schedule and Wednesday in the TTS schedule. Moreover, there were significant differences in the day of the week risks among the various CVD types. On trend analysis, the overall hospitalization rate for CVDs on the first HD day did not decrease (*P* = 0.2); however, the hospitalization rate for IDs on the first HD day significantly decreased (*P* = 0.02) over a span of 20 years. When GEEs were used in the case of FHD patients with severe heart failure, the hospitalization rate on the first HD day (Monday) significantly decreased after FHD initiation (*P* = 0.04). It was found that HD schedules affected the day of the week of hospitalization for CVDs. FHD may lower the day of the week risk.

## Introduction

End-stage renal disease (ESRD) is associated with increased mortality and the risk of acquiring cardiovascular diseases (CVDs) [1]. In addition, the intermittency of hemodialysis (HD), which remains a major renal replacement therapy for ESRD patients, has led to an increase in CVD-associated mortality following the longest interdialytic gap due to the accumulation of body fluids, electrolytes, and uremic toxins [2–5].

In 1999, Bleyer *et al*. reported that sudden and cardiac death rates on Monday and Tuesday were higher in patients on the Monday-Wednesday-Friday (MWF) and Tuesday-Thursday-Saturday (TTS) HD schedules, respectively, using data from the United States Renal Data System (USRDS), over 20 years [2]. In the Dialysis Outcomes and Practice Patterns Study (DOPPS), conducted over 9 years, Zhang *et al*. also reported that CVD mortality was higher on Monday and Tuesday in patients on the MWF and TTS schedules, respectively [4]. According to an Australia and New Zealand Dialysis and Transplant (ANZDATA) Registry study, a daily variation in cardiac deaths were observed among those who received HD less than 3 time a week [6]. Moreover, Foley *et al*. [3] and Fotheringham *et al*. [5] described the association between hospitalization and HD schedules from the USRDS and UK Renal Registry (UKRR), respectively. However, these studies were conducted over relatively short time periods. It has also been noted that hospitalization rates and mortality related to infectious diseases (IDs) have been increasing in HD patients, according to a USRDS report [7] and a Japanese Society for Dialysis Therapy report [8], respectively.

Daily HD was first reported as a treatment option for acute renal failure in 1960 [9]; in the 1970s, frequent HD (FHD) was proposed as a treatment for uremia [10, 11]. It has been reported that daily HD increases heart rate variability [12], is associated with a decreased requirement for antihypertensive medications [13], and decreases hospitalization rates in ESRD patients [14]. Additionally, other studies showed that FHD improves the control of hypertension [15, 16] and mineral metabolism [17]; decreases sleep apnea, hypopnea, and hypoxemia [18]; reduces ventricular volumes [19]; and improves left ventricular (LV) mass [17, 20] and LV ejection fraction (LVEF) [20, 21].

However, no reports have evaluated the impact of HD schedules on the day of the week of hospitalization for CVDs and IDs in patients undergoing HD and FHD, over a period of time. In the present study, we examined the influence of the dialysis schedule on the day of the week of hospitalization for CVDs and IDs in HD patients, over a period of 20 years. We also evaluated the influence of FHD on the day of the week of hospitalization in HD patients with severe heart failure.

## Materials and methods

### Study design and population

This was a retrospective observational study of ESRD patients who received in-center HD at Tsuchiya General Hospital between January 1, 1995 and December 31, 2014. This study was approved by the Tsuchiya General Hospital Institutional Review Board for Human Investigation (approval number: E150518-4), and performed according to the principles of the Declaration of Helsinki. Written informed consent was waived due to the strict maintenance of patients’ anonymity and the observational nature of the study.

**Fig 1:**
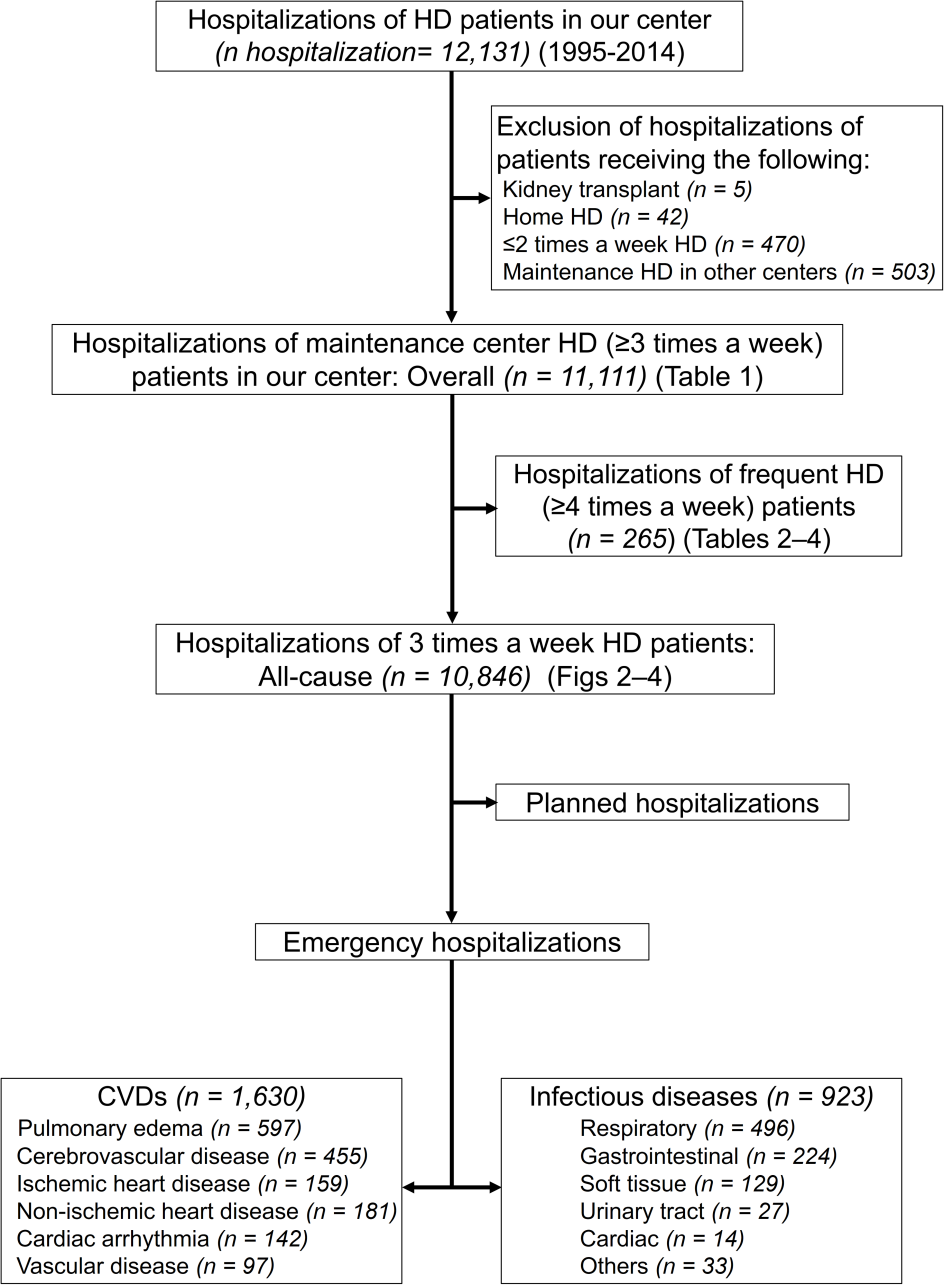
Hospitalization flow chart in hemodialysis patients, portraying the selection of the study population. CVDs: cardiovascular diseases; HD: hemodialysis.

We identified cases of hospitalization of ESRD patients who were 18 years or older and were receiving in-center HD at least 3 times a week (Fig 1). Cases of hospitalization were excluded if the patients received the following therapies: kidney transplants (5 hospitalizations), home HD (42 hospitalizations), HD not more than twice a week (470 hospitalizations), and maintenance HD at other centers including the change of hospitals (503 hospitalizations). Data were collected at dialysis initiation and included demographic characteristics such as age at dialysis initiation, sex, primary cause of ESRD, the day of hospitalization as well as the primary cause of hospitalization (defined by the International Classification of Diseases [ICD] 10 diagnosis code) [22]. As determined by our electronic medical data system and chart review, CVDs which led to emergency hospitalization were: pulmonary edema (PE) caused by exogenous fluid or congestive heart failure, cerebrovascular disease (CBVD), ischemic heart disease (IHD), non-IHD (including acute heart failure, cardiac arrest, hypertension, hypotension, shock, and valvular heart disease), cardiac arrhythmia, and aortic or peripheral vascular disease (VD). IDs which led to emergency hospitalization were: respiratory, gastrointestinal, soft tissue, urinary tract, and cardiac IDs, or other infections caused in a similar manner. The outpatient HD schedule was determined by the date of the HD prescription in the computer medical record system at our center. The MWF and TTS HD schedules were defined as the following: Monday and Tuesday, first HD day; Wednesday and Thursday, second HD day; Friday and Saturday, third HD day.

### Hemodialysis prescriptions

In our center, ESRD patients received HD through the use of high-flux membranes with the following dialysis doses: blood flow rate, 150–300 mL/min; and dialysate flow rate, 500 mL/min. The dialysate was composed of the following: sodium (Na^+^), 140 mEq/L; potassium (K^+^), 2.0 mEq/L; calcium (Ca^2+^), 2.75–3.0 mEq/L; magnesium (Mg^2+^), 1.0 mEq/L; chloride (Cl^-^), 110–112.25 mEq/L; acetate (CH_3_COO^-^), 0 or 8.0 mEq/L; Citrate^3-^, 0 or 2.0 mEq/L; bicarbonate (HCO_3_^-^), and 27.5–35.0 mEq/L; glucose (C_6_H_12_O_6_), 100–150 mg/dL. Body weight gain following the longest interdialytic gap was controlled within 6%. For those who received HD 3 times a week, HD was administered over 3–4 hours.

### Frequent HD

FHD was defined as HD at least 4 times a week: 4 or 5 times a week for 3–4 hours or 6 times a week for 2 hours. We initiated FHD for ESRD patients with severe heart failure, defined as per the New York Heart Association (NYHA) Functional Classification III or IV. In FHD patients, Monday was invariably the first HD day. The proportion of hospitalizations in FHD was calculated by dividing the number of hospitalizations in the patients receiving FHD by the total number of hospitalizations in the patients receiving HD at least 3 times a week during the same periods. Comparison between the groups was performed by generalized estimating equations (GEEs).

### Outcomes

The primary outcomes were the day of the week of hospitalization according to the MWF and TTS schedules, over a period of 20 years (1995–2014), as well as instances of hospitalization on each HD session day for CVDs and IDs, measured every 5 years (1995–99, 2000–04, 2005–09, 2010–14) in those who received HD 3 times a week. The secondary outcomes were the day of the week of hospitalization for CVDs and IDs in FHD patients.

### Statistical analysis

Data were analyzed using the Statistical Package for the Social Sciences version 14 (SPSS Inc., Chicago, IL, USA) and STATA version 14.2 (StataCorp., College Station, Texas, USA). The proportion of patients was presented based on the day of the week or the day of HD session hospitalization, and the median and interquartile range for such occurrences were also reported.

To examine differences in the demographic and hospitalization characteristics of patients as well as the day of the week distribution of hospitalization, GEEs with robust variance estimators were used. Some patients were hospitalized several times, and so, we used GEEs to consider correlations within the patients adequately [23, 24].

GEEs with robust variance estimators were used to identify the association between the day of the week and hospitalization for each type of CVD, in patients treated according to the MWF and TTS schedules. Covariates included in the model were the parameters of interest, including the day of the week of hospitalization, age, and dialysis vintage at admission. Instead of comparing each day of the week with a reference day, the odds of hospitalization for each type of CVD for a given day of the week were compared with the odds for the all days of the week.

To evaluate the time trend of hospitalization for CVDs and IDs according to HD sessions, stratified by 5-year intervals, hospitalization rates for CVDs and IDs on each HD session day were calculated and trend analyses were performed. Trend analysis performs the nonparametric test for trend across 5-year intervals, which is an extension of the Wilcoxon rank-sum test [25].

When GEEs were used to identify the independent predictors for hospitalizations on Monday in FHD patients, we analyzed hospitalizations both before and after FHD initiation in FHD patients. We considered “age at admission” as a continuous variable and “CVDs” (CVDs, 1; non-CVDs, 0), “IDs” (IDs, 1; non- IDs, 0), and “FHD” (receiving HD ≥4 times a week before hospitalization, 1; receiving HD 3 times a week before hospitalization, 0) as categorical variables. In all the analyses, *P-values* less than 0.05 were considered statistically significant.

## Results

### Patient and hospitalization characteristics

We analyzed 11,111 instances of hospitalization among 1,953 HD patients. The mean age (± SD) at dialysis initiation was 62.2 ± 14.4 years. Of the patients enrolled, 63.8% (*n* = 1,246) were male and 45.0% (*n* = 879) had diabetes mellitus (DM). The hospitalization flow chart for HD patients is shown in Fig 1. There were 10,846 hospitalizations among 1,936 patients receiving maintenance HD 3 times a week in our hospital, including emergency hospitalizations for CVDs (1,630 hospitalizations) and IDs (923 hospitalizations).

Patient and hospitalization characteristics among those who received HD at least 3 times a week are summarized in Table 1. The median age (interquartile) at admission increased every 5 years (1995–99, 61.6 [54.4–69.3] years; 2000–04, 67.0 [59.5–73.5]; 2005–09, 68.7 [61.1–76.1]; 2010–14, 71.2 [63.3–78.2] years [every *P* < 0.05, compared with 1995–99]). The proportion of patients with DM significantly increased, over the study period (1995–99, 36.3%; 2000–04, 42.6%; 2005–09, 47.0%; 2010–14, 50.8%; every *P* < 0.05, compared to 1995–99). The rate of CVDs contributing to all-cause hospitalization significantly decreased over the study period (1995–99, 32.9%; 2000–04, 18.6% [*P* < 0.001]; 2005–09, 13.0%; 2010–14, 9.6%; every *P* < 0.05, compared with 1995–99). The rate of IDs also decreased over the study period (1995–99, 11.8%; 2000–04, 8.5%; 2005–09, 8.3%; 2010–14, 7.7%; every *P* < 0.05, compared with 1995–99).

**Table 1:** Patient and hospitalization characteristics of patients who received hemodialysis at least 3 times a week.

Variable	Overall	1995–99	2000–04	2005–09	2010–14
**Number of hospitalizations, *n***	11,111	1,471	2,218	2,886	4,536
**Age at admission, *years*** **(interquartile)**	68.6 (60.5–75.9)	61.6 (54.4–69.3)	67.0* (59.5–73.5)	68.7* (61.1–76.1)	71.2* (63.3–78.2)
**Dialysis vintage at admission, *months* (interquartile)**	65.3 (28.3–123.0)	46.1 (19.8–98.1)	58.7* (28.4–106.5)	73.5* (34.5–122.1)	71.8* (28.6–137.0)
**Male, *n* (*%*)**	6,810 (61.3)	919 (62.5)	1,323 (59.7)	1,740 (60.3)	2,828 (62.4)
**Primary cause of ESRD**					
Diabetes mellitus, *n* (*%*)	5,136 (46.2)	534 (36.3)	945 (42.6)*	1,355 (47.0)*	2,302 (50.8)*
Chronic glomerulonephritis, *n* (*%*)	4,114 (37.0)	806 (54.8)	934 (42.1)*	1,061 (36.8)*	1,313 (29.0)*
Nephrosclerosis, *n* (*%*)	669 (6.0)	50 (3.4)	138 (6.2)*	158 (5.5)	323 (7.1)*
Polycystic kidney disease, *n* (*%*)	224 (2.0)	21 (1.4)	29 (1.3)	82 (2.8)	92 (2.0)
IgA nephropathy, *n* (*%*)	113 (1.0)	11 (0.8)	29 (1.3)	30 (1.0)	43 (1.0)*
Others, *n* (*%*)	267 (2.4)	24 (1.6)	83 (3.7)*	70 (2.4)	90 (2.0)
Unknown, *n* (*%*)	305 (2.8)	8 (0.5)	20 (0.9)	66 (2.3)*	211 (4.7)*
**Number of hospitalizations**					
**Total CVDs, *n* (*%*)**	1,704 (15.3)	484 (32.9)	413 (18.6)*	374 (13.0)*	433 (9.6)*
Pulmonary edema, *n* (*%*)	635 (5.7)	200 (13.6)	164 (7.4)*	117 (4.1)*	154 (3.4)*
Cerebrovascular disease, *n* (*%*)	466 (4.2)	85 (5.8)	117 (5.3)	125 (4.3)	139 (3.1)*
Ischemic heart disease, *n* (*%*)	174 (1.6)	34 (2.3)	36 (1.6)	49 (1.7)	55 (1.2)*
Non-ischemic heart disease, *n* (*%*)	185 (1.7)	87 (5.9)	47 (2.1)*	29 (1.0)*	22 (0.5)*
Cardiac arrhythmia, *n* (*%*)	144 (1.3)	45 (3.1)	32 (1.4)*	38 (1.3)*	29 (0.6)*
Vascular disease, *n* (*%*)	101 (0.9)	33 (2.2)	18 (0.8)*	16 (0.6)*	34 (0.8)*
**IDs, *n* (*%*)**	944 (8.5)	173 (11.8)	188 (8.5)*	235 (8.1)*	348 (7.7)*

Data are expressed as the median (interquartile range), numbers, and percentages for variables. Comparisons between groups were performed by generalized estimating equations. CVDs: cardiovascular diseases; ESRD: end-stage renal disease; IDs: infectious diseases; IgA, immunoglobulin A; SD: standard deviation.

*: *P* < 0.05 (compared with 1995–99)

Patient characteristics for those treated according to the MWF and TTS HD schedules are summarized in the S1 Table and S2 Table. Hospitalization characteristics for the MWF (6,013 hospitalizations) and TTS (4,833 hospitalizations) HD schedules are summarized in the S3 Table and S4 Table. Based on hospitalization schedules, the trend in hospitalization did not significantly differ over the 20-year study period.

### Distribution of day of week hospitalization in patients receiving HD 3 times a week

**Fig 2:**
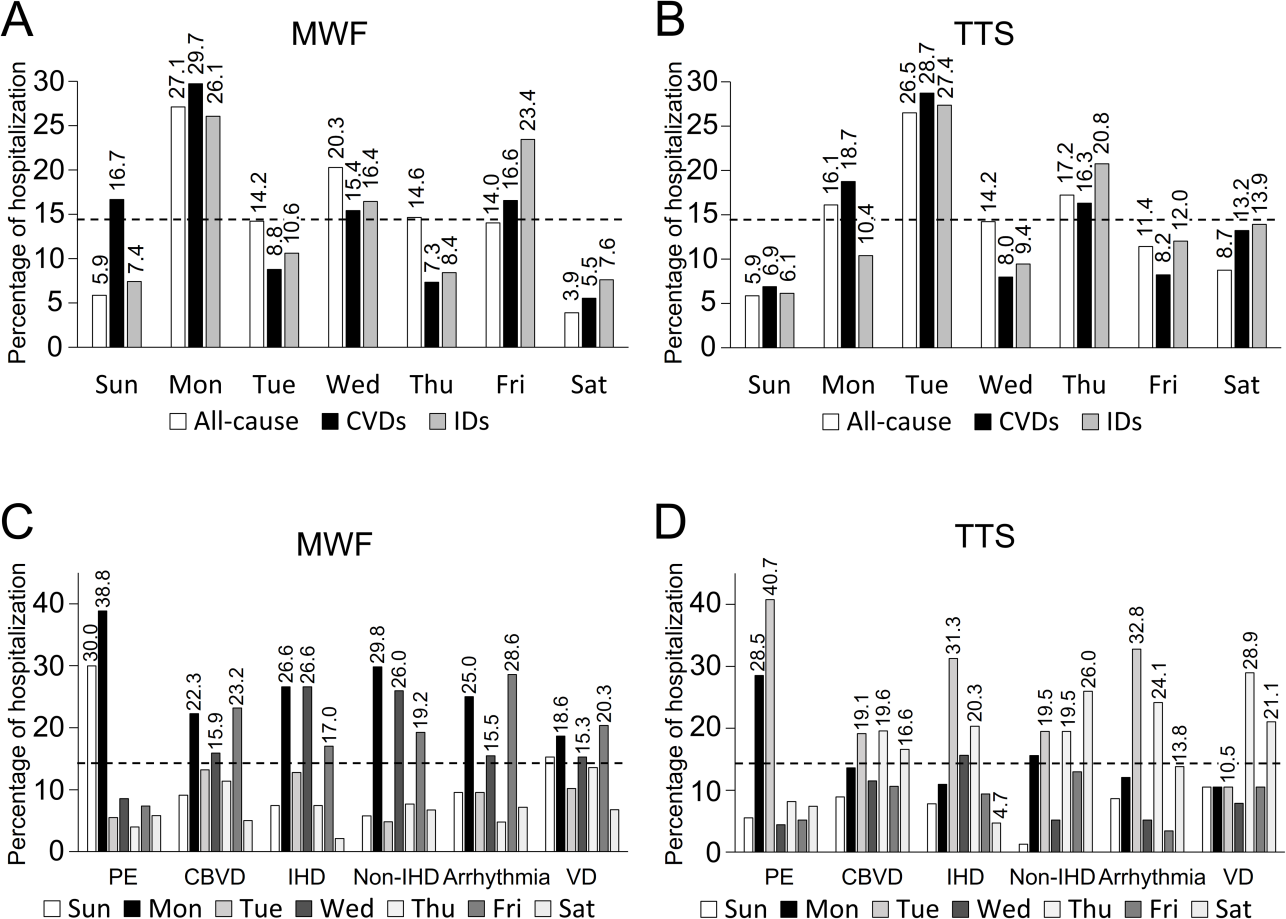
Distribution of hospitalization according to the day of the week of treatment for the Monday-Wednesday-Friday and Tuesday-Thursday-Saturday hemodialysis schedules. (A, B) All-cause, cardiovascular diseases, and infectious diseases in patients receiving hemodialysis in the (A) Monday-Wednesday-Friday and (B) Tuesday-Thursday-Saturday schedules. (C, D) Each type of cardiovascular disease in patients receiving hemodialysis in the (C) Monday-Wednesday-Friday and (D) Tuesday-Thursday-Saturday schedules. The dotted lines indicate the average rate of hospitalization on all the days of the week (1 out of 7 [14.3%]). CBVD: cerebrovascular disease; CVD: cardiovascular disease; IHD: ischemic heart disease; PE: pulmonary edema; VD: vascular disease.

Day of the week hospitalization rates in patients receiving HD on the MWF and TTS schedules are shown in Fig 2A and Fig 2B. Over the period of 20 years, hospitalization rates for all-cause hospitalization, CVDs, and IDs were the highest on Monday (27.1%, 29.7%, and 26.1%) in the MWF schedule (Fig 2A), and Tuesday (26.5%, 28.7%, and 27.4%) in the TTS schedule (both, first HD day) compared with the average rate of all the days of the week (1 out of 7 [14.3%]). The second highest hospitalization rates for CVDs were on Sunday (16.7%, *P* = 0.1) in the MWF schedule and Monday (18.7%) in the TTS schedule (both, third HD+2 days), compared with the average rate of all the days of the week (Fig 2B).

Day of the week hospitalization rates for each type of CVD are shown in Fig 2C and Fig 2D. For patients receiving HD on the MWF and TTS schedules, the rates of PE were the highest on Monday (38.8%) and Tuesday (40.7%), followed by Sunday (30.0%) and Monday (28.5%) compared with the average rate of all the days of the week (14.3%), respectively. For patients on both schedules, the rates of CBVD, IHD, non-IHD, and cardiac arrhythmia were higher on HD session days compared with the average rate of all the days of the week; however, this was not the same in cases of IHD and arrhythmia on Saturday, and VD on Tuesday, in the TTS schedule.

**Fig 3:**
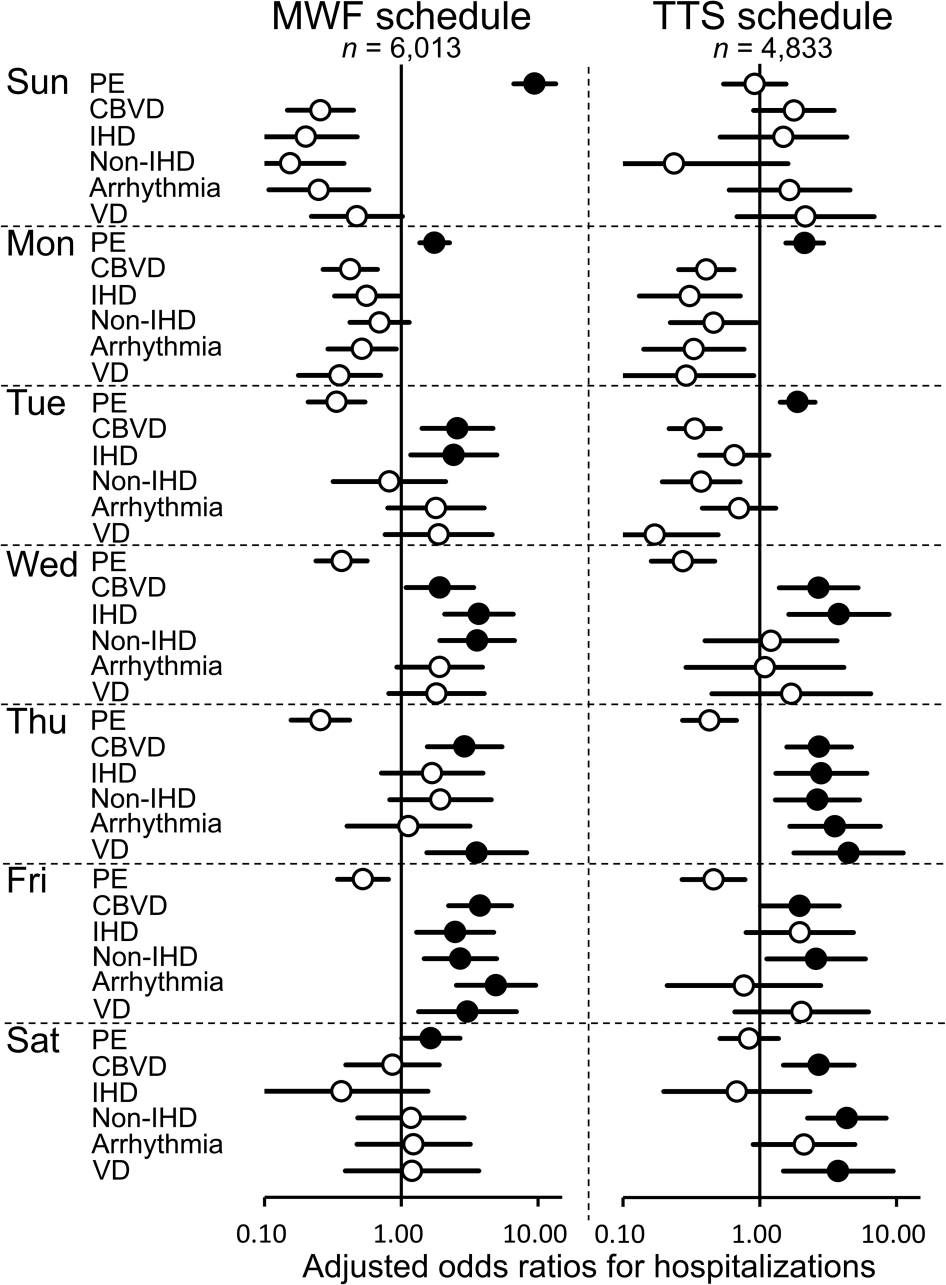
Generalized estimating equations of the day of the week as a predictor of hospitalization for each type of cardiovascular disease in the Monday-Wednesday-Friday and Tuesday-Thursday-Saturday hemodialysis schedules. The odds of hospitalization for each type of cardiovascular disease for a given day of the week were compared with those for all the days of the week. Analysis adjusted for age and dialysis vintage at admission. Markers are shown for variables (types of cardiovascular diseases) in each schedule. Shaded markers indicate statistical significantly higher ratios (*P* < 0.05). Absent markers indicate the variables that did not reach statistical significance. Error bars indicate 95% confidence intervals. CBVD: cerebrovascular disease; Fri: Friday; IHD: ischemic heart disease; Mon: Monday; PE: pulmonary edema; Thu: Thursday; Tue: Tuesday; Sat: Saturday; Sun: Sunday; Wed: Wednesday; MWF: Monday-Wednesday-Friday; TTS: Tuesday-Thursday-Saturday; VD: vascular disease.

On using GEEs with robust variance estimators, it was found that the day of the week was a significant independent predictor of hospitalization for each type of CVD, in each HD schedule (Fig 3). The odds of hospitalization for PE on the following days of the week were higher when compared to those for all the days of the week: Sunday (odds ratio [OR] 9.45, 95% confidence interval [CI] 6.65–13.44) and Monday (OR 1.75, 95% CI 1.36–2.25) in the MWF schedule; Monday (OR 2.13, 95% CI 1.55–2.94) and Tuesday (OR 1.89, 95% CI 1.41–2.54) in the TTS schedule. For CBVD and IHD, the odds of hospitalization on the following days of the week were higher when compared with those for all the days of the week: CBVD, Tuesday (OR 2.57, 95% CI 1.41–4.67) in the MWF schedule and Wednesday (OR 2.70, 95% CI 1.39–5.23) in the TTS schedule; IHD, Tuesday (OR 2.42, 95% CI 1.17–4.99) in the MWF schedule and Wednesday (OR 3.80, 95% CI 1.63–8.84) in the TTS schedule. For all types of CVDs except for PE, the odds of hospitalization on the second HD and third HD days of the week were predominantly higher when compared to those for all the days of the week, in both the MWF and TTS schedules.

**Fig 4:**
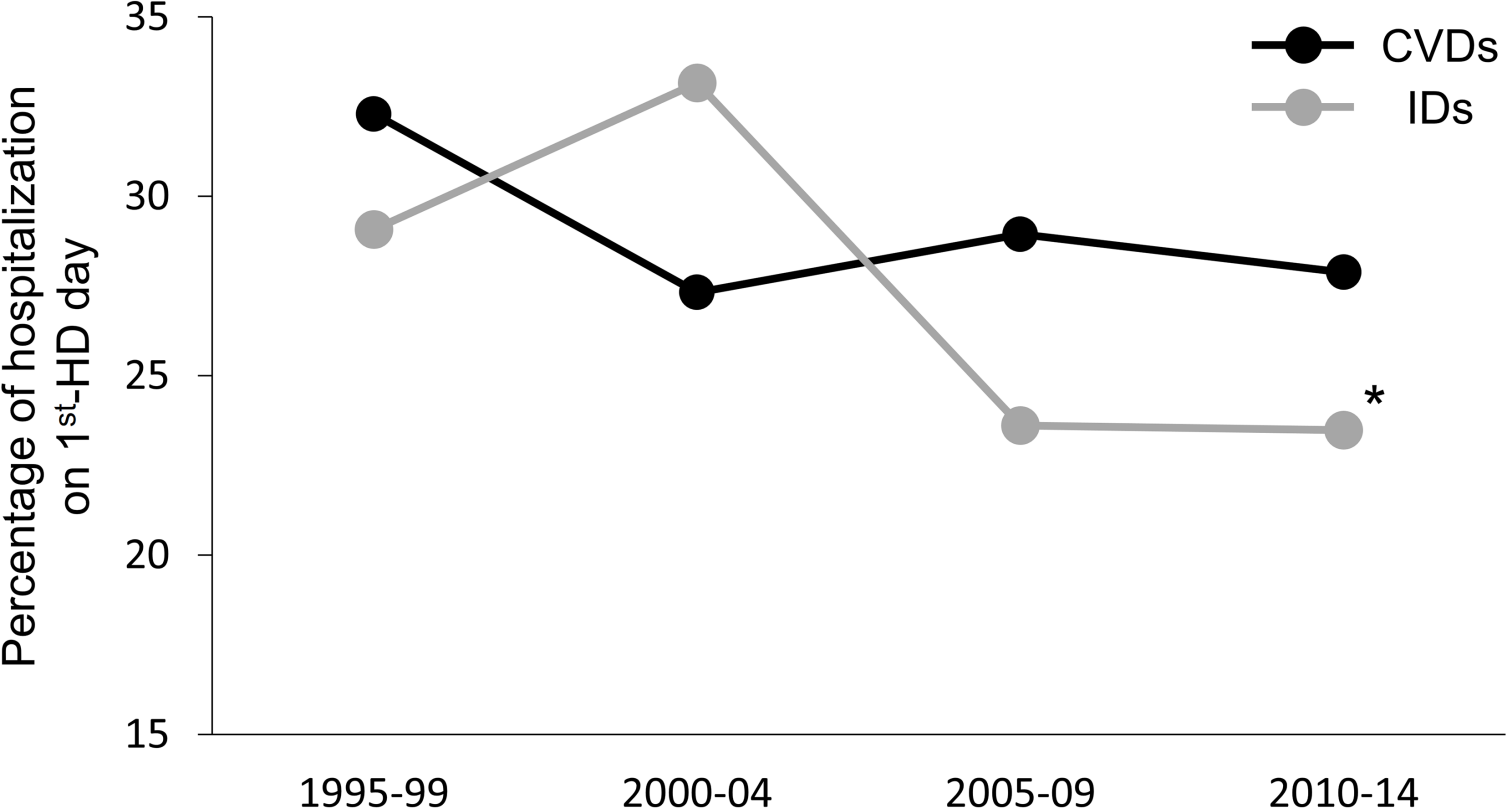
Time trend of proportion of hospitalization on the first HD day for cardiovascular and infectious diseases. The black line and markers indicate hospitalization rates for cardiovascular diseases on the first hemodialysis (HD) session days. The grey line and markers indicate hospitalization rates for infectious diseases on the first HD session days. CVDs: cardiovascular diseases; HD: hemodialysis; IDs: infectious diseases.

On trend analysis, the overall hospitalization rate for CVDs on the first HD day did not decrease over the 20 years (*P* = 0.2). However, the hospitalization rate for IDs on the first HD day significantly decreased over the course of 20 years (*P* = 0.02) (Fig 4).

### Hospitalization in frequent HD patients

Patient and hospitalization characteristics of all the patients who received FHD are summarized in Table 2. On comparing pre- and post-FHD initiation using GEEs, hospitalization rates for all-cause on Monday were found to be significantly reduced after FHD initiation (29.5% [150/509] versus 21.5% [57/265], *P* = 0.04). In addition, the hospitalization rates for CVDs on Monday were also reduced after FHD initiation, but there was no significant difference between them (36.3% [48/132] versus 25.7% [19/74], *P* = 0.1). However, the hospitalization rates for IDs on Monday were similar pre- and post-FHD initiation (16.1% [5/31] versus 14.3% [3/21], *P* = 0.9). A majority of the FHD patients received HD 4 times a week, but the frequency of HD provided 6 times a week increased during the study period (Table 3). On comparing the groups, using GEEs, the proportion of FHD patients was found to have increased in the last 10 years of the study (1995–99, 0.5%; 2005–09, 2.3% [*P* = 0.04]; 2010–14, 4.1% [*P* = 0.003]) (both *P*, compared with 1995–99).

**Table 2:** Patient and hospitalization characteristics of all the patients who received frequent hemodialysis.

Variable	Total N = 774	Pre-FHD N = 509	Post-FHD N = 265
**Age at admission, *years*** **(interquartile)**	63.1 (57.3–71.8)	62.1 (56.7–68.7)	66.6 (59.1–74.5)
**Dialysis vintage at admission, *months* (interquartile)**	64.3 (30.9–131.0)	54.9 (26.6–109.7)	89.6 (44.5–161.5)
**Male, *n* (*%*)**	523 (67.6)	321 (63.1)	202 (76.2)*
**Primary cause of end-stage renal disease**			
Diabetes mellitus, *n* (*%*)	293 (37.9)	190 (37.3)	103 (38.9)
Chronic glomerulonephritis, *n* (*%*)	377 (48.7)	251 (49.3)	126 (47.5)
Nephrosclerosis, *n* (*%*)	35 (4.5)	26 (5.1)	9 (3.4)
Polycystic kidney disease, *n* (*%*)	30 (3.9)	28 (5.5)	2 (0.8)
Immunoglobulin A nephropathy, *n* (*%*)	0 (0)	-	-
Others, *n* (*%*)	3 (0.4)	1 (0.2)	2 (0.8)
Unknown, *n* (*%*)	26 (3.4)	8 (1.6)	18 (6.8)
**Hospitalization for all-cause on Monday**, *n* (%)	207 (26.7)	150 (29.5)	57 (21.5)*

Data are expressed as the median (interquartile range), numbers, and percentages for variables. Comparisons between pre- and post-frequent HD initiation were performed by generalized estimating equations.

*: *P* < 0.05 (compared with pre-frequent HD).

CVDs: cardiovascular diseases; HD: hemodialysis; IDs: infectious diseases; SD: standard deviation.

**Table 3:** Hospitalizations in patients who received frequent hemodialysis.

Variable	1995–99	2000–04	2005–09	2010–14
**Number (proportion) of hospitalization in frequent HD patients, *n* (*%*)**	8 (0.5)	8 (0.4)	65 (2.3)*	184 (4.1)*
4 times a week HD, *n* (*%*)	8 (0.5)	7 (0.3)	42 (1.5)	139 (3.1)
5 times a week HD, *n* (*%*)	0 (0)	1 (0.05)	1 (0.03)	10 (0.2)
6 times a week HD, *n* (*%*)	0 (0)	0 (0)	22 (0.8)	35 (0.8)

The proportion of hospitalizations in frequent hemodialysis was calculated by dividing the number of hospitalizations in the patients receiving frequent hemodialysis by the total number of hospitalizations in the patients receiving hemodialysis at least 3 times a week during the same periods. Comparison between the groups was performed by generalized estimating equations.

*: *P* < 0.05 (compared with 1995–99).

HD: hemodialysis

When GEEs were used to identify the independent risk factors for hospitalizations on Monday, in all the patients who received FHD, FHD (OR 0.65, CI 0.44–0.94; *P* = 0.02) was a protective factor for hospitalization on Monday (first HD day) in the FHD patients (Table 4). However, the odds of CVDs (OR 1.42, CI 0.97–2.10; *P* = 0.07) for hospitalization on Monday in FHD patients was higher compared to the other factors, but this was not of statistical significance.

**Table 4:** Risk factors for hospitalizations on Monday among all patients who received frequent hemodialysis.

Variable	Hospitalizations on Monday		
	Odds ratio	95% CI	*P-value*
**Age at admission**	1.00	0.99–1.02	0.36
**CVDs**	1.42	0.97–2.10	0.07
**IDs**	0.54	0.26–1.12	0.10
**Frequent HD**	0.65	0.44–0.94	0.02

In the generalized estimation equations to identify the independent risk factors for hospitalizations on Monday in all the patients who received frequent hemodialysis, we analyzed hospitalizations both before and after frequent hemodialysis initiation, and considered “age at admission” as a continuous variable and “cardiovascular diseases” (cardiovascular diseases, 1; non-cardiovascular diseases, 0), “infectious diseases” (infectious diseases, 1; non-infectious diseases, 0), and “frequent hemodialysis” (≥4 times a week hemodialysis before hospitalization, 1; 3 times a week hemodialysis before hospitalization, 0) as categorical variables. CI: confidence interval; CVDs: cardiovascular diseases; HD: hemodialysis; IDs: infectious diseases

## Discussion

This study described the distribution of the day of the week of hospitalization for the various types of CVDs, based on HD schedules. Additionally, the influence of HD and FHD scheduling, on the day of the week of hospitalization for CVDs and IDs was investigated over a period of 20 years. Previous reports from the USRDS [3] and UKRR [5] demonstrated high hospitalization rates for CVDs following the long interdialytic interval; however, the time periods of these studies were only 4 and 5 years, respectively. There were also many variations in the treatment strategy and type of hospitalization (planned or emergency) among the institutions included in these studies.

In HD patients, the body fluids [26], various uremic toxins, and serum potassium [27] that accumulate over the longest interdialytic gap, may contribute to the day of week risk for CVDs. According to a previous report, exercise-induced afterload mismatch, assessed by the changes in potential pathophysiologic echocardiographic parameters, was most pronounced after the longest interdialytic gap [28]. Tsilonis *et al*. reported that the excess volume accumulation over the long interdialytic interval results in higher left and right arterial enlargement rates as well as right ventricular systolic pressure, on analysis of the echocardiographic parameters [29]. According to a DOPPS report, a relative interdialytic weight gain (IDWG) of at least 5.7% elevated the risk for mortality, and an IDWG of at least 4% elevated the risk for fluid-overload hospitalization [30]. In our study, hospitalizations for PE contributed to the high hospitalization rates for CVDs after the longest gap, in both the HD schedules (shown in Fig 2, Fig 3 and Fig 4). Our findings support the association between fluid overload and the day of week risk suggested in previous reports [26–30].

According to the DOPPS report by Zhang *et a*l., unexpectedly, Japanese patients on the MWF schedule had higher non-CVD mortality on Friday, and European patients on the TTS schedule had higher CVD mortality on Saturday [4]. The findings in European patients may be explained by the risks associated with HD therapy itself. In previous reports, intradialytic hypotension was found to be a risk factor of mortality [31, 32]. Moreover, Flythe *et al*. reported that higher ultrafiltration rates in HD are associated with a greater risk of all-cause and CVD mortality [33]. Therefore, excessive ultrafiltration may induce intradialytic hypotension in the HD sessions. In our study, the hospitalization risks for CBVD and IHD were higher on the day after the first HD session (shown in Fig 3). In addition, the hospitalization risks for all types of CVDs except for PE were predominantly higher on the second and third HD days (shown in Fig 3). Our findings support the risks of HD therapy suggested by previous reports [31–33].

Frequent nocturnal HD improved LV mass [34] and measures of mineral metabolism, and reduced blood pressure, compared to conventional HD [17, 35]. According to a USRDS report, nocturnal HD was associated with significant reductions in the risk of mortality and major morbid events compared to conventional HD [36]. The ANZDATA study reported that a daily variation in cardiac deaths was not observed in patients who underwent HD at least 4 times a week as well as peritoneal dialysis [6]. However, no previously done studies have examined the effect of the day of the week of hospitalization in before and after cases of FHD initiation, in ESRD patients with severe heart failure, according to the NYHA Functional Classification III or IV. In our study, we described that the rate of hospitalization on the first HD day decreased after FHD initiation, in cases of severe heart failure.

According to a USRDS report, infection-related mortality was higher following the longest interdialytic gap [3]. In another USRDS study, risk factors for infection-related hospitalizations were found to be age (higher risk with increasing age), sex (higher risk for females), history of diabetes, heart failure and pulmonary disease, as well as low serum albumin [37]. However, neither of these studies evaluated the day of the week risk for IDs. In our study, we described the day of the week of hospitalization for IDs, over a period of 20 years, and found that there were no significant differences in the day of the week of hospitalization for IDs, over the past 10 years (shown in Fig 4). Further studies are required to identify the day of the week risk for IDs.

This study had several limitations. First, our data did not include the exact time of admission or the timing of the dialysis sessions. We did not identify whether the patients were admitted before or after the dialysis sessions on the HD session days. Second, this study was based on a group of patients treated in a single hospital, and so the sample size was relatively small. Finally, in our study, FHD was not applied in a random fashion (FHD was initiated for patients with severe heart failure), thereby creating a bias which affects the conclusions that are to be drawn from this study. Because this is an observational study, confounding by indication may affect the association between FHD and hospitalization on Monday (Table 4). To control for confounding by indication, we included self-control (before and after FHD initiation) in our analysis using GEEs. We also adjusted for age at admission and cause of hospitalization. In spite of the use of these approaches, residual confounding should be considered and the interpretation of these results should be carried out with caution. However, since the analysis took place in a single hospital, it enabled us to advocate for the changes that should be implemented to the policies that surround the strategies for dialysis management. In addition, we were able to identify the exact emergency hospitalization rates for CVDs, because our data were confirmed by our electronic medical data system, chart review, as well as the ICD-10 code. Our study is valuable as no previous reports have assessed the influence of HD schedules on the day of the week of hospitalization for various types of CVDs, over a period of 20 years, and no studies have evaluated the day of the week risk of hospitalization before and after FHD initiation.

## Conclusions

HD schedules affected hospitalization for CVDs, but not for IDs. FHD may lower the day of the week risk of hospitalization. Our findings can help inform clinicians in selecting the dialysis modality as well as guide clinical management practices for CVDs and IDs in ESRD patients.

## Supporting information

S1 Table Characteristics of patients treated according to the Monday-Wednesday-Friday hemodialysis schedule. (DOCX)

S2 Table Characteristics of patients treated according to the Tuesday-Thursday-Saturday hemodialysis schedule. (DOCX)

S3 Table Hospitalization characteristics of hemodialysis patients in the Monday-Wednesday-Friday schedule. (DOCX)

S4 Table Hospitalization characteristics of hemodialysis patients in the Tuesday-Thursday-Saturday schedule. (DOCX)

S1 File Hospitalization and patient data in patients receiving in-center hemodialysis at least 3 times a week. We identified cases of hospitalization of end-stage renal disease patients who were 18 years or older and were receiving in-center maintenance hemodialysis at least 3 times a week at Tsuchiya General Hospital between January 1, 1995 and December 31, 2014.
(XLSX)
