# Supplementary material for: The impact of hemodialysis schedules on the day of the week of hospitalization for cardiovascular and infectious diseases, over a period of 20 years
Source: PLoS One. 2017 Jul 10;12(7):e0180577. doi: 10.1371/journal.pone.0180577 (PMC5503277; doi:10.1371/journal.pone.0180577)
Supplement: S1 Table — (DOCX) [file pone.0180577.s001.docx]

**S1 Table. Characteristics of patients treated according to the Monday-Wednesday-Friday hemodialysis schedule**

| **Variable** | **Overall**  ***n* = 6,013** | **1995-99**  ***n* = 756** | **2000-04**  ***n* = 1,251** | **2005-09**  ***n* = 1,622** | **2010-14**  ***n* = 2,384** |
| --- | --- | --- | --- | --- | --- |
| **Age at admission, *years***  **(interquartile)** | 67.9  (60.0-75.1) | 62.3  (54.8-70.1) | 66.4  (59.7-73.1) | 67.9  (59.8-75.7) | 70.1  (62.6-77.7) |
| **Dialysis vintage at admission, *months***  **(interquartile)** | 73.5  (32.8-129.9) | 50.9  (20.3-112.8) | 62.3  (32.7-99.6) | 85.1  (40.5-127.1) | 83.5  (33.0-147.6) |
| **Male, *n* (*%*)** | 3,742 (62.2) | 482 (63.8) | 813 (65.0) | 1,012 (62.4) | 1,435 (60.2) |
| **Primary cause of ESRD** |  |  |  |  |  |
| Diabetes mellitus, *n* (*%*) | 2,762 (45.9) | 270 (35.7) | 526 (42.1) | 740 (45.6) | 1,226 (51.4) |
| CGN, *n* (*%*) | 2,230 (37.1) | 416 (55.0) | 502 (40.1) | 601 (37.1) | 711 (29.8) |
| Nephrosclerosis, *n* (*%*) | 383 (6.4) | 28 (3.7) | 108 (8.6) | 81 (5.0) | 166 (7.0) |
| PCK, *n* (*%*) | 170 (2.8) | 14 (1.9) | 25 (2.0) | 71 (4.4) | 18 (3.0) |
| IgA nephropathy, *n* (*%*) | 66 (1.1) | 5 (0.7) | 16 (1.3) | 17 (1.1) | 28 (1.2) |
| Others, *n* (*%*) | 115 (1.9) | 5 (0.7) | 40 (3.2) | 44 (2.7) | 26 (1.1) |
| Unknown, *n* (*%*) | 127 (2.1) | 4 (0.5) | 9 (0.7) | 28 (1.7) | 86 (3.6) |

Data are expressed as the median (interquartile range), numbers, and percentages for variables. CGN: chronic glomerulonephritis; ESRD: end-stage renal disease; IgA: immunoglobulin A; PCK: polycystic kidney disease; SD: standard deviation.
