# Supplementary material for: The impact of hemodialysis schedules on the day of the week of hospitalization for cardiovascular and infectious diseases, over a period of 20 years
Source: PLoS One. 2017 Jul 10;12(7):e0180577. doi: 10.1371/journal.pone.0180577 (PMC5503277; doi:10.1371/journal.pone.0180577)
Supplement: S2 Table — (DOCX) [file pone.0180577.s002.docx]

**S2 Table. Characteristics of patients treated according to the Tuesday-Thursday-Saturday hemodialysis schedule**

| **Variable** | **Overall**  ***n* = 4,833** | **1995-99**  ***n* = 707** | **2000-04**  ***n* = 959** | **2005-09**  ***n* = 1,199** | **2010-14**  ***n* = 1,968** |
| --- | --- | --- | --- | --- | --- |
| **Age at admission, *years***  **(interquartile)** | 69.5  (61.3-76.9) | 61.1  (54.3-68.7) | 67.4  (59.0-74.5) | 70.0  (62.4-77.2) | 73.2  (65.1-79.2) |
| **Dialysis vintage at admission, *months***  **(interquartile)** | 55.7  (23.3-111.6) | 41.9  (19.2-82.2) | 54.2  (23.3-117.2) | 57.9  (26.8-107.3) | 59.8  (23.9-119.6) |
| **Male, *n* (*%*)** | 2,866 (59.3) | 431 (61.0) | 509 (53.1) | 685 (57.1) | 1,241 (63.1) |
| **Primary cause of ESRD** |  |  |  |  |  |
| Diabetes mellitus, *n* (*%*) | 2,271 (47.0) | 263 (37.2) | 418 (43.6) | 593 (49.5) | 997 (50.7) |
| CGN, *n* (*%*) | 1,756 (36.3) | 383 (54.2) | 426 (44.4) | 424 (35.4) | 523 (26.6) |
| Nephrosclerosis, *n* (*%*) | 277 (5.7) | 22 (3.1) | 29 (3.0) | 73 (6.1) | 153 (7.8) |
| PCK, *n* (*%*) | 52 (1.1) | 7 (1.0) | 4 (0.4) | 10 (0.8) | 31 (1.6) |
| IgA nephropathy, *n* (*%*) | 47 (1.0) | 6 (0.9) | 13 (1.4) | 13 (1.1) | 15 (0.8) |
| Others, *n* (*%*) | 150 (3.1) | 19 (2.7) | 43 (4.5) | 26 (2.2) | 62 (3.2) |
| Unknown, *n* (*%*) | 160 (3.3) | 4 (0.6) | 11 (1.2) | 37 (3.1) | 108 (5.5) |

Data are expressed as the median (interquartile range), numbers, and percentages for variables. CGN: chronic glomerulonephritis; ESRD: end-stage renal disease; IgA: immunoglobulin A; PCK: polycystic kidney disease; SD: standard deviation.
