# Supplementary material for: The impact of hemodialysis schedules on the day of the week of hospitalization for cardiovascular and infectious diseases, over a period of 20 years
Source: PLoS One. 2017 Jul 10;12(7):e0180577. doi: 10.1371/journal.pone.0180577 (PMC5503277; doi:10.1371/journal.pone.0180577)
Supplement: S3 Table — (DOCX) [file pone.0180577.s003.docx]

**S3 Table. Hospitalization characteristics of hemodialysis patients in the Monday-Wednesday-Friday schedule**

| **Variable** | **Overall**  ***n* = 6,013** | **1995-99**  ***n* = 756** | **2000-04**  ***n* = 1,251** | **2005-09**  ***n* = 1,622** | **2010-14**  ***n* = 2,384** |
| --- | --- | --- | --- | --- | --- |
| **CVDs, *n* (*%*)** | 888 (14.8) | 246 (32.5) | 232 (18.6) | 205 (12.6) | 205 (8.6) |
| Pulmonary edema, *n* (*%*) | 327 (5.4) | 89 (11.8) | 104 (8.3) | 61 (3.8) | 73 (3.1) |
| Cerebrovascular disease, *n* (*%*) | 220 (3.7) | 51 (6.8) | 52 (4.2) | 61 (3.8) | 56 (2.4) |
| Ischemic heart disease, *n* (*%*) | 95 (1.6) | 15 (2.0) | 22 (1.8) | 25 (1.5) | 33 (1.4) |
| Non-ischemic heart disease, *n* (*%*) | 104 (1.7) | 50 (6.6) | 25 (2.0) | 19 (1.2) | 10 (0.4) |
| Cardiac arrhythmia, *n* (*%*) | 84 (1.4) | 18 (2.4) | 21 (1.7) | 30 (1.9) | 15 (0.6) |
| Vascular disease, *n* (*%*) | 59 (1.0) | 23 (3.0) | 9 (0.7) | 9 (0.6) | 18 (0.8) |
|  |  |  |  |  |  |
| **Infectious diseases, *n* (*%*)** | 499 (8.3) | 92 (12.2) | 93 (7.4) | 127 (7.8) | 187 (7.8) |

Data are expressed as the numbers and percentages for variables. CVDs: cardiovascular diseases; IDs: infectious diseases.
