# Supplementary material for: The impact of hemodialysis schedules on the day of the week of hospitalization for cardiovascular and infectious diseases, over a period of 20 years
Source: PLoS One. 2017 Jul 10;12(7):e0180577. doi: 10.1371/journal.pone.0180577 (PMC5503277; doi:10.1371/journal.pone.0180577)
Supplement: S4 Table — (DOCX) [file pone.0180577.s004.docx]

**S4 Table. Hospitalization characteristics of hemodialysis patients in the Tuesday-Thursday-Saturday schedule**

| **Variable** | **Overall**  ***n* = 4,833** | **1995-99**  ***n* = 707** | **2000-04**  ***n* = 959** | **2005-09**  ***n* = 1,199** | **2010-14**  ***n* = 1,968** |
| --- | --- | --- | --- | --- | --- |
| **CVDs, *n* (*%*)** | 742  (15.4) | 234  (33.1) | 178  (18.6) | 144  (12.0) | 186  (9.5) |
| Pulmonary edema, *n* (*%*) | 270 (5.6) | 107 (15.1) | 57 (5.9) | 45 (3.8) | 61 (3.1) |
| Cerebrovascular disease, *n* (*%*) | 235 (4.9) | 34 (4.8) | 65 (6.8) | 60 (5.0) | 76 (3.9) |
| Ischemic heart disease, *n* (*%*) | 64 (1.3) | 19 (2.7) | 14 (1.5) | 19 (1.6) | 12 (0.6) |
| Non-ischemic heart disease, *n* (*%*) | 77 (1.6) | 37 (5.2) | 22 (2.3) | 8 (0.7) | 10 (0.5) |
| Cardiac arrhythmia, *n* (*%*) | 58 (1.2) | 27 (3.8) | 11 (1.2) | 7 (0.6) | 13 (0.7) |
| Vascular disease, *n* (*%*) | 38 (0.8) | 10 (1.4) | 9 (0.9) | 5 (0.4) | 14 (0.7) |
|  |  |  |  |  |  |
| **IDs, *n* (*%*)** | 424 (8.8) | 80 (11.3) | 94 (9.8) | 106 (8.8) | 144 (7.3) |

Data are expressed as the numbers and percentages for variables. CVDs: cardiovascular diseases; IDs: infectious diseases.
